# From Critical Thinking to Passive Acceptance: The Moderating Role of Decision Fatigue in Students’ Use of Generative AI

**DOI:** 10.3390/ejihpe16090130

**Published:** 2026-08-29

**Authors:** Marco Zuin, Vanessa Donadel

**Affiliations:** 1 Department of Psychology, Istituto Universitario Salesiano Venezia, 30174 Venice, Italy; 2 Department of Business & Management, Libera Università Internazionale degli Studi Sociali “Guido Carli”, 00197 Rome, Italy; vdonadel@external.luiss.it

**Keywords:** critical thinking, decision fatigue, Generative Artificial Intelligence, students, higher education

## Abstract

Academic research on Artificial Intelligence (AI) has primarily focused on usage motivations, AI literacy, trust, overreliance, and critical use. However, the mechanisms through which critical thinking influences passive reliance on AI-generated output in academic contexts remain largely unexplored. This study examined the role of Critical Thinking and Decision Fatigue in students’ passive acceptance of AI-generated output. A sample of 383 Italian university students completed an online survey including the Critical Thinking Disposition Scale and the Decision Fatigue Scale. Of these, 332 students reported having used GenAI in the previous three months and were presented with six task-specific items assessing reliance on unmodified AI-generated output across six academic tasks (Idea generation, Text generation, Text revision, Creating quizzes, Receiving feedback, and Information search). Multiple hierarchical regression analyses tested the moderating role of Decision Fatigue. Decision Fatigue was not a significant direct predictor but moderated the relationship between Critical Thinking and passive acceptance in several academic activities, with the strength of this evidence varying according to the applied correction for multiple comparisons. The findings highlighted the role of critical thinking as a protective filter against the passive use of generative AI. However, this disposition is not always available, particularly under conditions of cognitive fatigue. The study offers new educational insights into the need to design instructional interventions that foster critical thinking while promoting cognitively sustainable learning environments.

## 1. Introduction

Generative Artificial Intelligence is widely used by university students to complete academic tasks. Indeed, across several studies, approximately 70% to 90% of students report using generative AI tools, such as ChatGPT, for study-related activities (Almassaad et al., 2024; Hirabayashi et al., 2024; Xiao et al., 2025).

This high rate of adoption has raised concerns regarding the impact that the systematic use of this technology for academic tasks (e.g., information search and synthesis, text and idea generation) may have on students’ cognitive development, potentially creating conditions of overreliance and hindering a critical and informed approach to managing the information provided by AI systems (Danaher, 2018; Shanmugasundaram & Tamilarasu, 2023; Jošt et al., 2024; Zhai et al., 2024; Abubakar et al., 2025; Sarwar et al., 2026).

Critical thinking is recognized as one of the key competencies of the twenty-first century for academic success and personal development (Rusmin et al., 2024). Traditionally, critical thinking has been defined by Ennis (1987) as a process of reasonable reflective thinking that guides judgment and action. Subsequently, Ennis (1989) introduced the evaluative dimension of critical thinking, emphasizing that it should guide individuals in analyzing the information available to them and foster a disposition to approach tasks with reflective skepticism. This latter aspect introduces a central component of critical thinking, namely the distinction between disposition and ability (Facione, 1990). Perkins et al. (1993) defined dispositions as patterns of intellectual behavior that shape and guide thinking and action, arguing that abilities alone are insufficient to produce intelligent behavior. Building on this perspective, the authors conceptualized disposition as an integrated system comprising inclination (the natural tendency to engage in a particular type of behavior), sensitivity (the ability to recognize when a situation requires the activation of that behavior), and abilities (the actual capacity to enact it). With regard to critical thinking in the context of interaction with GenAI, this perspective suggests that critical thinking extends beyond the expression of cognitive abilities. Rather, it encompasses an inclination to question the information provided by the system, a sensitivity to recognize situations in which scrutiny is required, and the actual ability to critically evaluate information by comparing it with other sources and with one’s own knowledge. In this sense, although critical thinking dispositions may represent relatively general tendencies, their expression appears to be closely related to the specific cognitive demands of the task (Abrami et al., 2015).

Melisa et al. (2025) conducted a systematic review on the impact of AI use on critical thinking. The authors observed that, on the one hand, ChatGPT can support the development of critical thinking by assisting students in analyzing and evaluating information more efficiently. On the other hand, however, there is a risk that students may accept information provided by ChatGPT without verifying its reliability, a practice that could weaken their ability to evaluate and process information critically. According to the authors (Melisa et al., 2025), the effect of Generative Artificial Intelligence on critical thinking depends on how individuals use this technology, starting from the prompts they generate (e.g., requesting the system to produce a text in place of the individual vs. requesting feedback on one’s own work) to the way they evaluate and use the generated output (e.g., critical evaluation of the information vs. passive acceptance). Oliva-Córdova et al. (2025) designed workshops incorporating the use of GenAI with the aim of fostering the development of critical thinking and observed significant improvements in the subdimensions of applying, analyzing, and evaluating, which are associated with higher-order thinking activities.

Within this context, much of the existing literature has examined how the use of generative AI may influence the development of students’ critical thinking (Essien et al., 2024; Tahir et al., 2024; Tian & Zhang, 2025; Gonsalves, 2026). The present study adopts a complementary perspective: rather than considering critical thinking as an outcome of interaction with technology, it is conceptualized as a dispositional characteristic that precedes such interaction. Critical thinking, conceptualized by Sosu (2013) as critical openness and reflective skepticism, may shape the way students interact with generative AI by influencing their tendency either to critically evaluate the system-generated output or to accept it more passively. This dynamic is embedded within a broader process of cognitive effort reorganization. As highlighted by Lee et al. (2025), artificial intelligence does not eliminate the need for critical thinking but rather changes its nature by shifting cognitive effort from task execution to supervision. Specifically, three main changes can be observed. In knowledge and comprehension tasks, cognitive effort shifts from information gathering to information verification, requiring the ability to identify errors. In application tasks, the focus moves from autonomous problem-solving to the critical integration of AI-generated output into the specific context and task. In processes involving analysis, synthesis, and evaluation, there is a transition from performing the task to managing it, with the individual assuming responsibility for guiding and evaluating the quality of the generated output while maintaining responsibility for the final outcome.

The way in which Generative Artificial Intelligence is used is determined not only by individual dispositions but also by the individual’s cognitive state and the availability of cognitive resources. In this regard, the construct of Decision Fatigue (DF; Hickman et al., 2018) is particularly relevant, as it describes a state of cognitive fatigue resulting from repeated decision-making that may reduce the capacity to exercise control, evaluation, and reflective monitoring. The concept of DF is based on the assumption that individuals are capable of making only a limited number of high-quality decisions within a given period of time, beyond which the quality of the decision-making process and the effectiveness of judgment begin to deteriorate (Al-Hammouri et al., 2024), with consequences for both self-control and motivation (Grignoli et al., 2025).

Applied to interaction with generative AI, reduced cognitive resources may affect the extent to which individuals are able to enact their critical thinking dispositions when evaluating AI-generated output. Thus, DF may be particularly relevant not necessarily as a direct determinant of passive acceptance, but as a condition that may alter the relationship between Critical Thinking and the tendency to accept AI-generated output without substantial modification.

Related evidence comes from research examining different forms of cognitive and mental fatigue in the context of AI use.

Shalu et al. (2025), for example, found that prolonged interaction with artificial intelligence systems was associated with information overload and mental exhaustion, as well as lower self-confidence in decision-making, suggesting that sustained interaction with AI may be accompanied by cognitive states that can affect how individuals engage with information and make decisions. The authors also reported weak and, in some cases, inverse associations between different dimensions of AI interaction and fatigue, suggesting that the psychological experience of AI engagement is multifaceted. Similarly, Khan and Shehawy (2025) examined decision fatigue in relation to cognitive load and found that these conditions were associated with response biases and impaired decision-making processes, with implications for the way individuals evaluate AI-supported decisions. These findings suggest that cognitive strain may compromise users’ capacity to critically evaluate AI-mediated decisions.

Evidence from related digital contexts points in a similar direction. Ahmed and Rasul (2023) conducted a study on Social Media Fatigue (SMF), defined as a state of cognitive exhaustion caused by social media use. Although SMF is conceptually distinct from the Decision Fatigue, their findings provide related evidence regarding the role of cognitive exhaustion in the evaluation of online information. The authors found that SMF was not directly associated with misinformation sharing in most of the contexts examined. Rather, the indirect effects suggested that higher SMF was associated with greater sharing through individuals’ perceptions of misinformation as accurate. These findings suggest that fatigue may influence information-related behaviour through evaluative processes rather than necessarily producing a direct behavioural effect. Moreover, higher cognitive ability acted as a protective factor by reducing belief in fake news. Although these studies address different forms of fatigue and different contexts, together they suggest that states of cognitive exhaustion may influence the extent to which individuals engage in reflective evaluation of externally generated information.

This perspective is particularly relevant to the present study because Critical Thinking represents a dispositional tendency toward reflective and evaluative processing, whereas Decision Fatigue reflects a state that may affect the cognitive resources available to enact such a disposition. Accordingly, rather than assuming that Decision Fatigue directly increases passive reliance on GenAI, we proposed that it may condition the extent to which Critical Thinking is translated into active evaluation of AI-generated information, thereby affecting the tendency to accept AI-generated output without substantial modification or verification.

Several studies have highlighted how uncritical reliance on artificial intelligence systems can compromise the quality of cognitive processes, learning, and achieved outcomes. Buçinca et al. (2021) observed that users tend to rely on suggestions provided by traditional AI systems (explainable AI) even when these prove to be incorrect, yielding lower performance compared to those who complete the task independently. The authors attribute this phenomenon both to the cognitive cost associated with verifying information generated by AI and to the tendency to interpret system-generated explanations as a signal of competence, leading to a reduced activation of analytical thinking and control processes. Similar findings emerge in educational settings. Bastani et al. (2025) demonstrated that unrestricted access to GPT Base encourages the direct copying of answers without a genuine understanding of the content. Although this mode of use improves performance during practice activities, students who utilized the system achieved significantly worse results on final examinations (conducted without AI support) compared to the control group. Furthermore, the authors highlight a discrepancy between perceived and actual learning, suggesting that students are not fully aware of the negative effects stemming from excessive reliance on the system. Comparable evidence has also been observed in professional contexts. Dell’Acqua et al. (2026) found that workers using AI to solve a managerial task were less likely to produce correct solutions than those working without technological support, likely due to overreliance on system-generated responses and a reduction in critical oversight activities. In line with these results, Gerlich (2025) observed a strong negative correlation between the use of AI tools and critical thinking, contending that reduced engagement in evaluation, analysis, and verification activities may contribute, over the long term, to the weakening of critical thinking and foundational cognitive skills.

Overall, this body of evidence suggests that the use of GenAI does not inevitably entail negative consequences; rather, risks emerge when users passively accept system-generated outputs without engaging in adequate verification, monitoring, and critical evaluation processes. It therefore becomes fundamental to understand which individual characteristics may foster or counteract this tendency, with particular reference to those cognitive and self-regulatory processes that support the critical evaluation of information generated by AI systems, thereby promoting usage that supports learning without replacing the cognitive processes required for knowledge construction.

**Figure 1:**
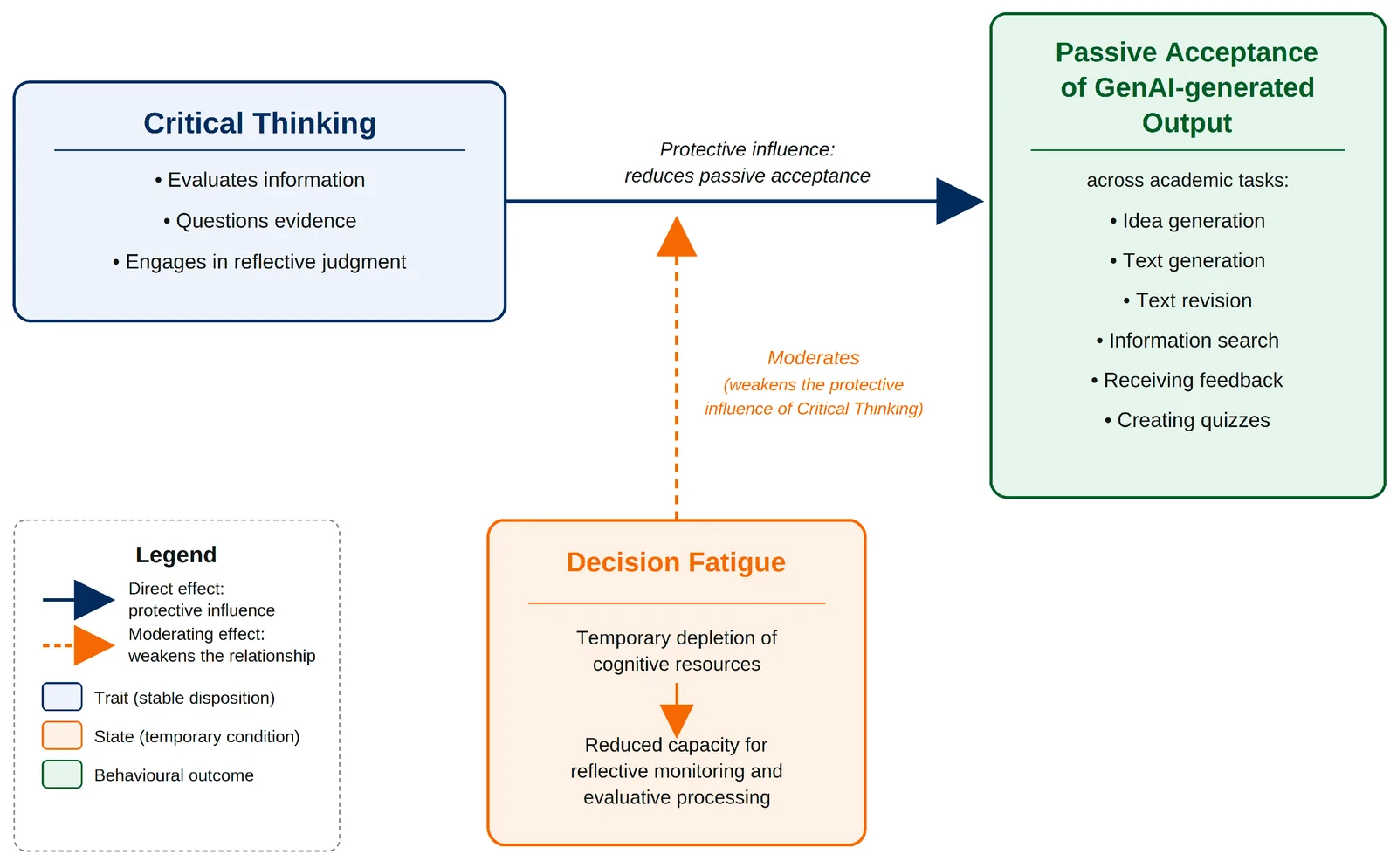
Theoretical model linking trait-level Critical Thinking to Passive Acceptance of GenAI output, moderated by state-level Decision Fatigue.

Building on these theoretical perspectives, the present study proposes an integrated explanatory framework linking Critical Thinking, Decision Fatigue, and the passive acceptance of GenAI-generated output (Figure 1). Critical Thinking is conceptualized as a relatively stable disposition that predisposes individuals to critically evaluate information, question evidence, and engage in reflective judgment before accepting externally generated content (Sosu, 2013). Under ordinary cognitive conditions, this disposition should reduce the likelihood of passively relying on AI-generated outputs without verification or personal revision. However, the behavioural expression of critical thinking is not expected to depend solely on dispositional characteristics, but also on the availability of cognitive resources. Decision Fatigue, as a temporary state of cognitive resource depletion (Hickman et al., 2018), may reduce individuals’ capacity to sustain reflective monitoring and evaluative processing during interaction with GenAI. Consequently, even students with a strong disposition toward critical thinking may be less able to translate this disposition into critical behaviour when experiencing high levels of Decision Fatigue. Within this framework, passive acceptance of GenAI-generated output is interpreted as the behavioural consequence of the interaction between a stable cognitive disposition (Critical Thinking) and a transient cognitive state (Decision Fatigue), whereby decision fatigue weakens the protective influence of critical thinking by limiting the cognitive resources required for reflective evaluation across different academic tasks.

The present study aims to investigate the role of critical thinking in the degree of reliance on GenAI, defined as the passive acceptance of AI-generated output. Specifically, it seeks to examine whether this protective resource is consistently available to individuals, thereby safeguarding them against the inappropriate use of the technology, or whether there are conditions under which, despite possessing high levels of critical thinking, individuals nonetheless passively accept AI-generated output.

Recent studies have identified several variables that mediate or moderate reliance on GenAI, including self-efficacy (Zhang et al., 2024; Acosta-Enriquez et al., 2025; Jia et al., 2025), self-control (Sun et al., 2026), perseverance (Galindo-Domínguez et al., 2026), AI literacy (Tian & Zhang, 2025; Pitts et al., 2026), cognitive offloading (Miranda et al., 2025), and cognitive fatigue (Tian & Zhang, 2025).

In the present study, Decision Fatigue is instead identified as the moderating variable, with the hypothesis that different levels of Decision Fatigue moderate the relationship between critical thinking and the passive acceptance of GenAI-generated output.

Within this framework, the present study addresses the following research hypotheses:

**H1.**  *Higher levels of Critical Thinking are associated with a lower tendency to use generative AI output without personal modification or revision.*

**H2.**  *Decision Fatigue is not expected to show a consistent direct association with the passive acceptance of GenAI-generated outputs across academic tasks.*

**H3.**  *Decision Fatigue moderates the relationship between Critical Thinking and the tendency to use generative AI output without personal modification or revision.*

## 2. Materials and Methods

### 2.1. Data Collection and Sample

The study adopted a cross-sectional quantitative design aimed at examining the psychological predictors of the passive acceptance (i.e., without personal modification) of AI-generated output across different academic tasks. Specifically, the study focused on the association between individual differences in critical thinking disposition and decision fatigue and the tendency to rely on generative AI when performing cognitive tasks.

The participants were 383 Italian university students enrolled in undergraduate and master’s degree programs (273 females and 110 males; mean age = 22.0 years, SD = 2.41).

Participants were recruited through convenience sampling by disseminating an online survey link via informal channels, including social media and student WhatsApp groups, among students enrolled at 35 Italian universities (with the largest groups of participants enrolled at IUSVE, the University of Padua, the University of Milano-Bicocca, and the University of Udine; see Appendix B for the complete list). The data collection was coordinated by IUSVE, with no formal collaboration with other institutions.

Participants’ academic disciplines were classified as STEM or non-STEM following the disciplinary classification adopted by Uddin et al. (2021). Based on this classification, 98 participants (25.6%) were enrolled in STEM disciplines, including engineering, computer science, mathematics, whereas 285 (74.4%) were enrolled in non-STEM disciplines, predominantly psychology, education, communication, and sociology.

A total of 383 students completed the survey. All participants answered a screening question assessing whether they had used GenAI during the previous three months. Of these, 332 participants (86.7%) reported recent GenAI use and were subsequently presented with the task-specific questions, whereas 51 (13.3%) who reported no GenAI use were not presented with the task-specific questions. Among eligible GenAI users, the number of responses varied across the six task-specific items: generating ideas (n = 295), generating text (n = 265), text revision (n = 258), creating quizzes (n = 212), receiving feedback (n = 229), and information search (n = 308). Participants who indicated that they had never used GenAI for a given activity were excluded only from the corresponding analysis.

To assess whether the available sample size was adequate for the planned analyses, an a priori power analysis was conducted using G*Power 3.1 for a hierarchical multiple regression model including six predictors: sex, age, STEM field, Critical Thinking, Decision Fatigue, and the Critical Thinking × Decision Fatigue interaction term. Assuming a medium effect size (*f*^2^ = 0.15), α = 0.05, and statistical power of 0.95, the analysis indicated a minimum required sample size of N = 146. All task-specific regression models exceeded this threshold. A convenience sampling strategy was adopted. Data were collected between March and April 2026. Participation was voluntary, and no incentives were provided.

Data were collected through an online questionnaire administered via Google Forms. Participants completed the questionnaire individually and remotely. A self-generated identification code was used to screen responses for potential duplicate submissions; no duplicate responses were identified. Before accessing the survey, participants were presented with an information sheet describing the aims of the study, the research procedures, confidentiality safeguards, and data protection measures. Informed consent was obtained electronically. The research protocol was approved by the Institutional Ethics Committee (IUSVE-EC-2026-002) and was conducted in accordance with the European Union General Data Protection Regulation (EU 2016/679; GDPR) and the ethical principles of the Declaration of Helsinki (1964) and its subsequent revisions.

The survey instrument consisted of three sections. The first section included six task-specific items developed to assess the frequency with which participants used unmodified generative AI output across different academic activities. The second section included standardized psychometric scales measuring critical thinking disposition (Sosu, 2013) and decision fatigue (Hickman et al., 2018). The final section collected participants’ socio-demographic information.

### 2.2. Instruments

#### 2.2.1. Critical Thinking Disposition Scale (CTDS)

Critical thinking disposition was assessed using the 11-item Critical Thinking Disposition Scale (CTDS; Sosu, 2013), rated on a 5-point Likert scale ranging from 1 (Strongly disagree) to 5 (Strongly agree). In his theoretical model, Sosu conceptualizes critical thinking as a multidimensional construct integrating two fundamental components: a cognitive dimension, referring to reasoning and analytical abilities, and a dispositional dimension, referring to the habitual tendency to engage in reflective and evaluative thinking. The CTDS specifically assesses this dispositional component.

The scale comprises two subdimensions: Critical Openness, which refers to the tendency to be receptive to new ideas, consider alternative perspectives, and revise one’s views when necessary (e.g., “It’s important to understand other people’s viewpoint on an issue”); and Reflective Skepticism, which refers to the tendency to question evidence, critically evaluate information, and reflect on previous experiences before making judgments (e.g., “I usually check the credibility of the source of information before making judgements”). In addition to the subscale scores, the CTDS allows the calculation of an overall dispositional score representing a general propensity toward critical thinking.

In the present study, the instrument was administered in Italian. Permission to translate the scale was obtained from the original author, and the translation was carried out following established cross-cultural adaptation procedures to ensure conceptual and linguistic equivalence (Beaton et al., 2000; Cruchinho et al., 2024). The Italian validation is currently underway; preliminary analyses conducted on an independent stratified sample of 418 participants indicated satisfactory internal consistency (Cronbach’s α = 0.69; McDonald’s ω = 0.69), supporting the use of the Italian version for research purposes while the validation study is ongoing. Although these values fall slightly below the conventional 0.70 benchmark commonly recommended for research applications, reliability coefficients in the 0.60–0.70 range have been considered acceptable in exploratory research and during the early stages of scale validation (Hair et al., 2019, p. 122).

#### 2.2.2. Decision Fatigue Scale (DFS)

Decision fatigue was assessed using the Decision Fatigue Scale (DFS) developed by Hickman et al. (2018). The scale measures subjective decision fatigue, conceptualized as a state of cognitive and emotional dysregulation resulting from mental exhaustion and impaired self-regulation, which may reduce the quality of decision-making.

The instrument consists of nine items (e.g., “It is hard for me to take in information and use it to make decisions”) rated on a 4-point Likert scale ranging from 0 (Strongly disagree) to 3 (Strongly agree). The total score is calculated by summing the responses to all items, with higher scores indicating greater perceived decision fatigue.

The DFS was originally developed as a unidimensional measure designed to capture the combined influence of emotional distress, cognitive depletion, and impulsive decision-making tendencies.

For the purposes of the present study, the instrument was administered in Italian. Permission to translate the scale was obtained from the original author, and the translation was carried out following established cross-cultural adaptation procedures to ensure conceptual and linguistic equivalence (Beaton et al., 2000; Cruchinho et al., 2024). The Italian validation is currently underway; preliminary analyses conducted on an independent stratified sample of 533 participants indicated good internal consistency (Cronbach’s α = 0.84; McDonald’s ω = 0.84), supporting the use of the Italian version for research purposes while the validation study is ongoing.

#### 2.2.3. Reliance on AI-Generated Output

To assess students’ reliance on AI-generated output, six task-specific items were developed. Each item referred to a specific academic activity (Table 1) and asked participants to indicate how frequently they relied on AI-generated content without making any personal modifications. The academic activities were identified based on the relevant literature (Johnston et al., 2024; Stojanov et al., 2024; Wang et al., 2025) and included: Idea generation (brainstorming), Text generation (e.g., writing an essay or a thesis chapter), Text revision (of a text written by the user; e.g., punctuation and spelling), Creating quizzes (or personalized exercises), Receiving feedback (on a text produced by the user), and Information search. No formal expert panel review or pilot testing was conducted prior to administration. An example item is: “In the past 3 months, when using GenAI for text generation (e.g., writing an essay or a thesis chapter), how often do you use the generated output without making substantial revisions?”.

Responses were recorded on a 5-point Likert scale ranging from 1 (Never) to 5 (Always), with higher scores indicating a greater tendency to rely on AI-generated output without making personal modifications or revisions. Each item also included an additional response option: “I have never used GenAI for this activity”. This option was included because students may have used generative AI for academic purposes in some activities but not in others.

Before completing these items, participants were asked a screening question: “Have you used generative AI for study-related activities during the past three months?” Only participants who responded affirmatively were directed to the six activity-specific items. This screening question was introduced to ensure that responses were based on recent and relevant experiences, thereby reducing recall bias and increasing the ecological validity of the measure. Thus, the screening question assessed participants’ overall experience with GenAI use in academic contexts, whereas the additional item-level response option (“I have never used GenAI for this activity”) allowed participants to indicate that they had not used GenAI for a specific activity.

From a methodological perspective, this distinction was essential to avoid conflating a low level of reliance with the absence of exposure. Treating non-use as equivalent to “Never” on the frequency scale would have introduced bias into the subsequent statistical analyses, as “Never” referred to never relying on AI-generated output without personal modification for that specific activity, rather than never having used GenAI for that activity. Designing the task-specific items to account for these different situations was intended to improve construct validity and preserve the interpretability of activity-specific reliance patterns.

It is important to note that these six items were not intended to function as interchangeable indicators of a single latent construct, nor were they designed to form a psychometric scale requiring internal consistency estimation (e.g., Cronbach’s alpha). Rather, each item was developed to capture a conceptually and behaviorally distinct academic activity in which passive acceptance of AI-generated output may vary according to the specific cognitive demands involved.

Table 1 presents the descriptive statistics for the six items. The number of respondents differs across activities because responses indicating “I have never used GenAI for this activity” were treated as missing data (structurally missing data).

### 2.3. Data Analysis

Hierarchical multiple regression was used to examine whether the level of Decision Fatigue moderated the relationship between Critical Thinking and the use of AI-generated output without personal modification or revision. Each academic activity, represented by one of the six task-specific items assessing passive acceptance of GenAI-generated output (see Table 1), was entered as a separate dependent variable.

Statistical analyses were performed using IBM SPSS Statistics (Version 29), the PROCESS macro for SPSS (Version 3.5), and Jamovi (Version 2.7.24).

Due to the task-specific nature of the items, participants provided responses only for AI-related activities they had actually performed. Consequently, sample size varied across regression models, as non-response reflected the absence of engagement in a given AI-related task rather than conventional missing data. Although effective sample sizes differed across tasks, all regression models were estimated on samples generally considered adequate for multiple regression analyses with a limited number of predictors. Therefore, despite task-specific differences in effective sample size, the available data were considered adequate for testing the planned hierarchical regression models and examining whether the interaction between Critical Thinking and Decision Fatigue contributed to the prediction of each AI-related task.

The analyses followed a hierarchical modeling strategy. Model 1 included the control variables: sex (0 = female, 1 = male), age, and university field (0 = non-STEM, 1 = STEM). Model 2 added Critical Thinking and Decision Fatigue. Finally, Model 3 included the interaction term between Critical Thinking and Decision Fatigue (CT × DF).

Regarding the interpretation of the hierarchical regression models, it is common practice (Hayes, 2022, pp. 547–548) to first enter the independent variable and the moderator and interpret the regression coefficients obtained at this stage (Model 2 in the present study), before introducing the interaction term to determine whether this interpretation should be qualified in light of evidence that the effect of the independent variable on the dependent variable is contingent upon the moderator (Model 3 in the present study). Consistent with the recommendations of Hayes (2022), the present study focuses its interpretation on the conditional nature of the effect of X on Y, examining the interaction through the analysis of conditional effects. The individual direct effects of the independent variable and the moderator should be interpreted in Model 2, because in Model 3, following the inclusion of the interaction term, they no longer represent partial effects but rather conditional effects, that is, effects estimated under the assumption that the other variable is equal to zero (Hayes, 2022, p. 244).

With regard to the specification of the regression models, Critical Thinking (independent variable) and Decision Fatigue (moderator) were neither standardized nor mean-centered. This decision was motivated by the structure of the dependent variable, which contained structurally missing data resulting from participants having no prior experience with a specific AI-supported academic activity, as indicated by the response option “I have never used GenAI for this activity”. Consequently, standardizing the variables using the full sample would not have constituted a methodologically appropriate procedure, because the variables entered into the analyses would not have been genuinely standardized with respect to the sample used in each regression model (Hayes, 2022, pp. 335–336). Specifically, each regression was estimated on a subset of the overall sample, comprising only participants who had previously used GenAI for the corresponding academic activity. The same methodological limitation would also apply to mean-centering.

Hierarchical linear regression was adopted as the primary analytical framework, as this approach is well established in psychological and educational research involving Likert-type outcomes with moderately large samples, and parametric methods have been shown to be generally robust when applied to such data under typical research conditions (Norman, 2010; Sullivan & Artino, 2013). This framework also allowed the moderation hypotheses to be specified and tested using Hayes’ PROCESS analytical approach (Hayes, 2022), which is widely employed in psychological research for estimating and interpreting interaction effects.

Given that the outcome variables were measured on 5-point Likert scales, ordinal logistic regression represents a valid and appropriate alternative for these data. To evaluate whether the substantive conclusions depended on this analytical choice, all six moderation models were additionally re-estimated using ordinal logistic regression, with likelihood-ratio comparisons between the main-effects model (Model 2) and the interaction model (Model 3) used as the primary criterion for evaluating the interaction term, given its closer correspondence to the model-comparison logic adopted in the linear regression analyses. Results of this robustness analysis are reported in Appendix A.

Because Hypothesis 3 was tested six times, once for each academic activity, the six interaction *p*-values were additionally submitted to three multiple comparison correction procedures differing in stringency: Bonferroni (Dunn, 1961), Holm (1979), and the Benjamini–Hochberg False Discovery Rate procedure (FDR; Benjamini & Hochberg, 1995). All three procedures are reported alongside the uncorrected *p*-values to allow evaluation of the robustness of the interaction effects under corrections of differing conservativeness.

## 3. Results

The hierarchical regression models described in Section 2.3 identified a significant interaction term (CT × DF) at the uncorrected α = 0.05 level for the following academic activities: Text revision, Creating quizzes, Receiving feedback, and Information search (Table 2). Table 3 reports the results of the Bonferroni, Holm, and FDR corrections applied to these interaction terms, together with the two non-significant terms (Idea generation, Text generation), for comparison.

To interpret the direction of these interactions, simple slope analyses and their corresponding significance tests were conducted (Table 4).

Predicted levels of passive acceptance of GenAI-generated output (for each academic activity) were examined as a function of Critical Thinking across three levels of Decision Fatigue: low (one standard deviation below the mean), moderate (the mean), and high (one standard deviation above the mean).

**Figure 2:**
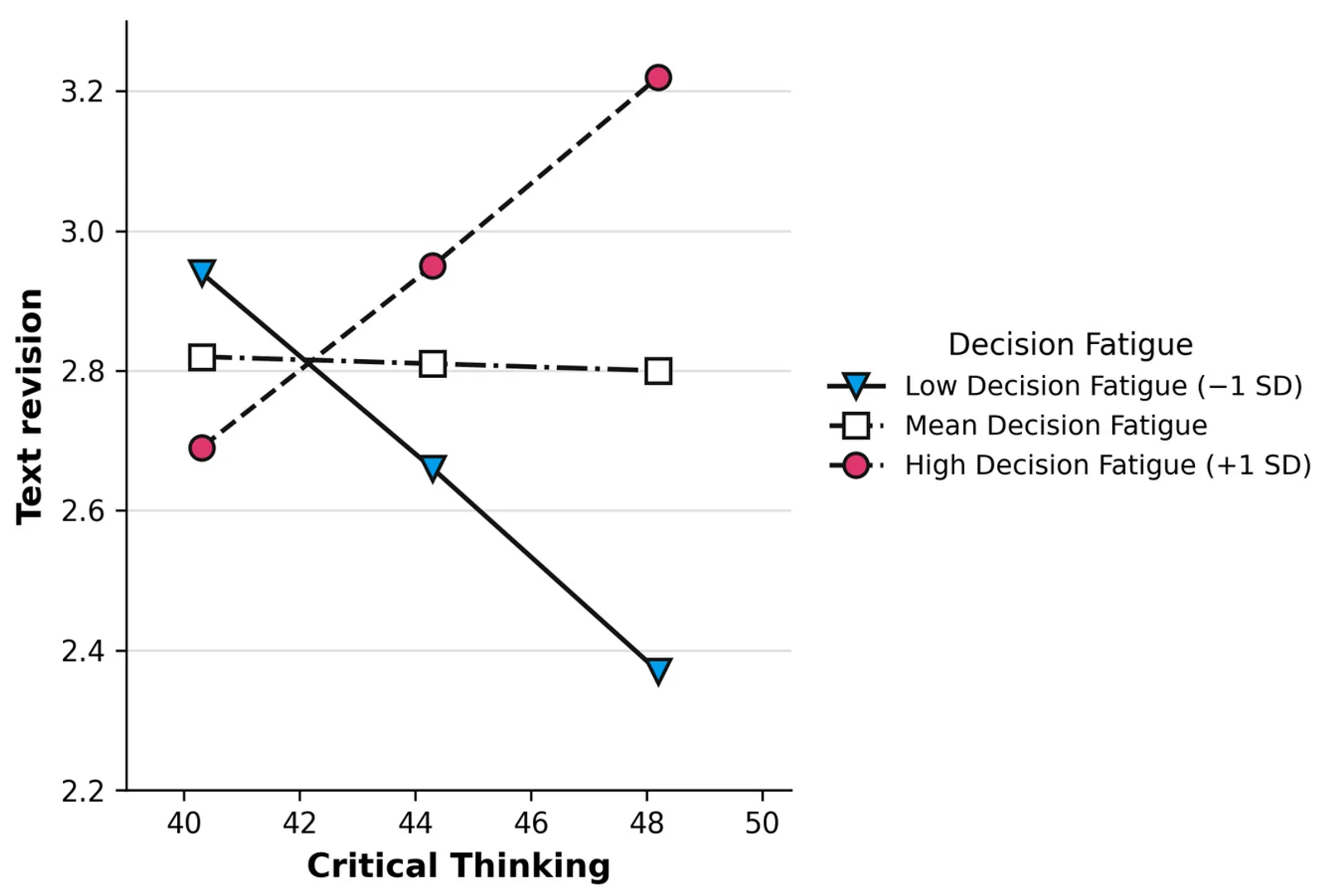
Moderating effect of Decision Fatigue on the relationship between Critical Thinking and passive acceptance of GenAI Output for Text revision.

With regard to Text revision as the dependent variable, no statistically significant predictors emerged in Models 1 or 2. In Model 3, the interaction term between Critical Thinking and Decision Fatigue was introduced. This addition resulted in a significant increase in explained variance (ΔR^2^ = 0.054, F = 14.50, *p* < 0.001, df = 251). The final model was statistically significant (*p* = 0.008), explaining 6.7% of the variance in the dependent variable. The interaction between Critical Thinking and Decision Fatigue was significant, indicating that the relationship between critical thinking and the passive acceptance of AI-generated output in the Text revision task varies as a function of the level of decision fatigue. The conditional effects analysis (Figure 2) showed that, at low levels of Decision Fatigue (−1 SD), Critical Thinking was associated with a significant reduction in the passive acceptance of AI-generated output (b = −0.072, *p* = 0.005). In contrast, at high levels of Decision Fatigue (+1 SD), Critical Thinking was associated with a significant increase in the passive acceptance of AI-generated output (b = 0.067, *p* = 0.016). These findings suggest that Decision Fatigue significantly moderates the role of critical thinking by influencing its protective effect against the passive acceptance of AI-generated output. This interaction remained significant after Bonferroni, Holm, and FDR correction for multiple comparisons (Table 3), indicating a robust effect.

**Figure 3:**
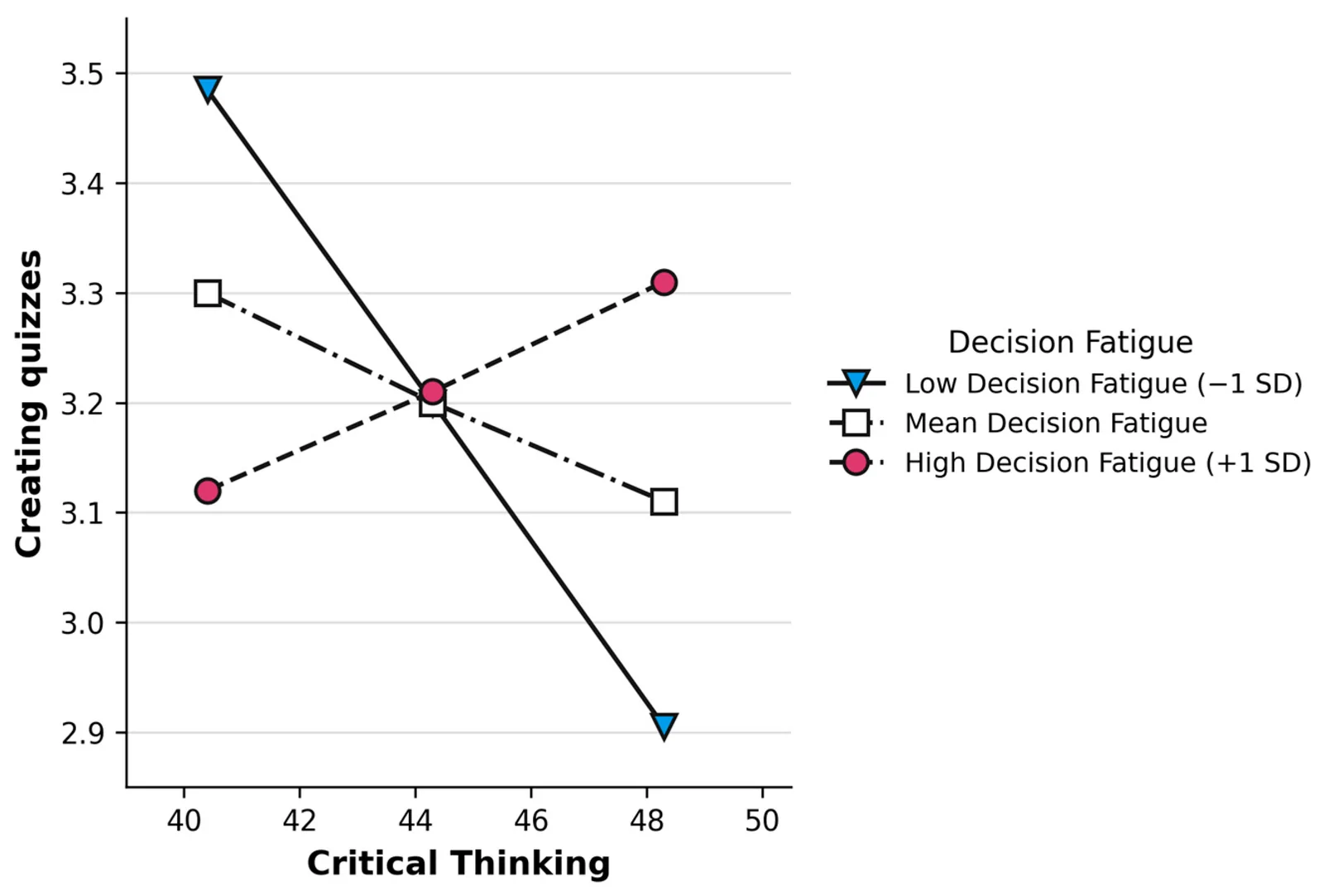
Moderating effect of Decision Fatigue on the relationship between Critical Thinking and passive acceptance of GenAI Output for Creating quizzes.

With regard to Creating quizzes as the dependent variable, Models 1, 2, and 3 were all statistically significant. Model 3 accounted for an additional 2.2% of the explained variance (ΔR^2^ = 0.022, F = 3.39, df = 205), resulting in a total explained variance of 9.0%. In Models 1 and 2, age was the only significant predictor, showing a significant negative effect. In Model 3, the interaction between Critical Thinking and Decision Fatigue was statistically significant (b = 0.010, SE = 0.004, t = 2.22, *p* = 0.028). This effect remained significant under FDR correction but did not survive Bonferroni or Holm correction (Table 3), and should therefore be interpreted with caution. The conditional effects analysis (Figure 3) showed that, at low levels of Decision Fatigue (−1 SD), Critical Thinking was associated with a significant reduction in the passive acceptance of AI-generated output (*p* = 0.027) in the Creating quizzes activity. These findings indicate that age has a direct negative effect on the dependent variable, such that increasing age is associated with a lower tendency to accept GenAI-generated output without making personal modifications or revisions. Furthermore, the moderation analysis indicates that, under conditions of low Decision Fatigue, higher levels of Critical Thinking are associated with a lower tendency to passively accept GenAI-generated output when creating quizzes.

**Figure 4:**
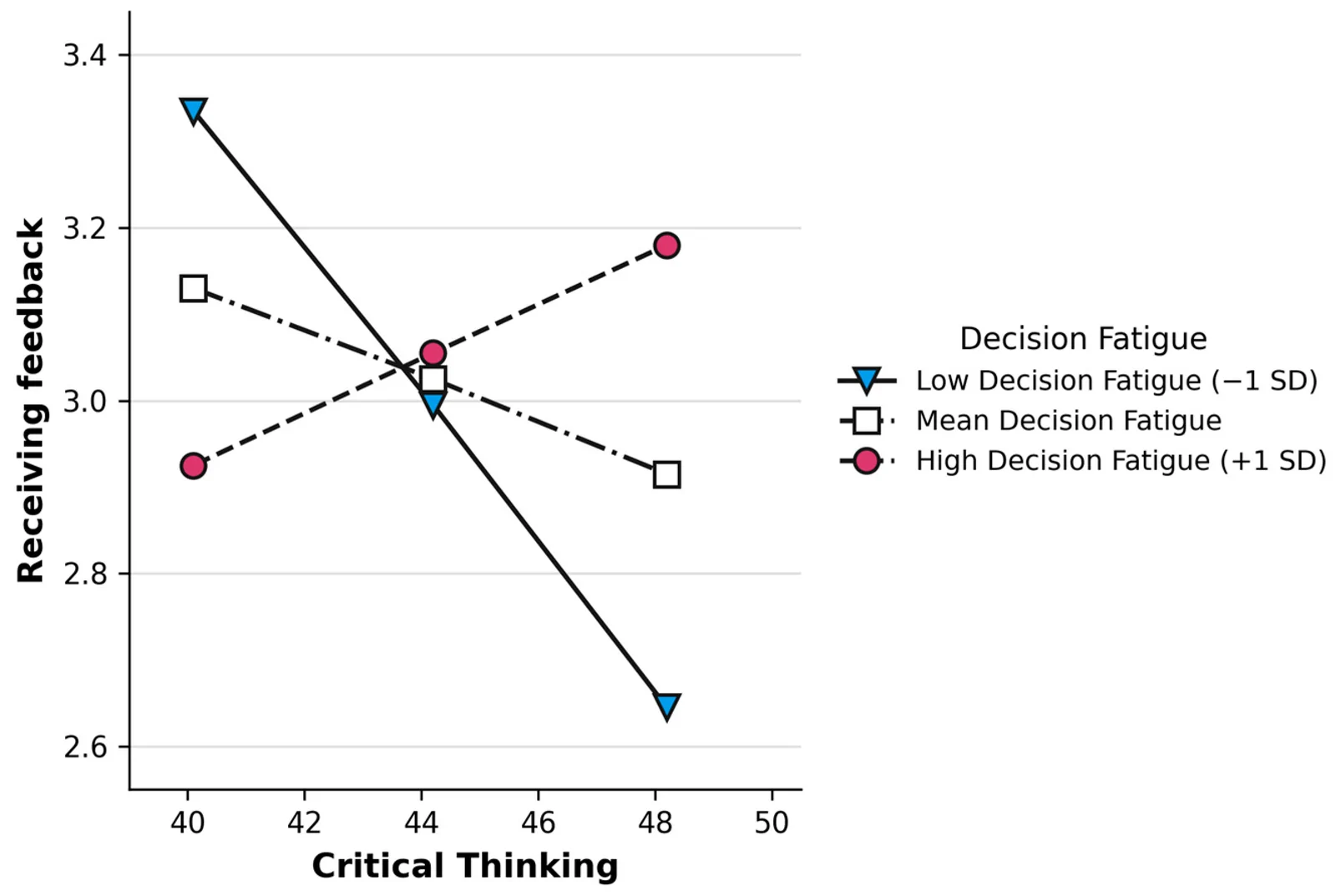
Moderating effect of Decision Fatigue on the relationship between Critical Thinking and passive acceptance of GenAI Output for Receiving feedback.

For the analysis with Receiving feedback as the dependent variable, only the final model (Model 3) was statistically significant (ΔR^2^ = 0.040, F = 2.48, *p* = 0.024, df = 222), explaining a total of 6.3% of the variance. This interaction effect remained significant after Bonferroni, Holm, and FDR correction (Table 3). The conditional effects analysis (Figure 4) showed that, at low levels of Decision Fatigue (−1 SD), Critical Thinking was associated with a significant reduction in the passive acceptance of AI-generated output (b = −0.085, *p* = 0.002) in the Receiving feedback activity involving participants’ own written work.

**Figure 5:**
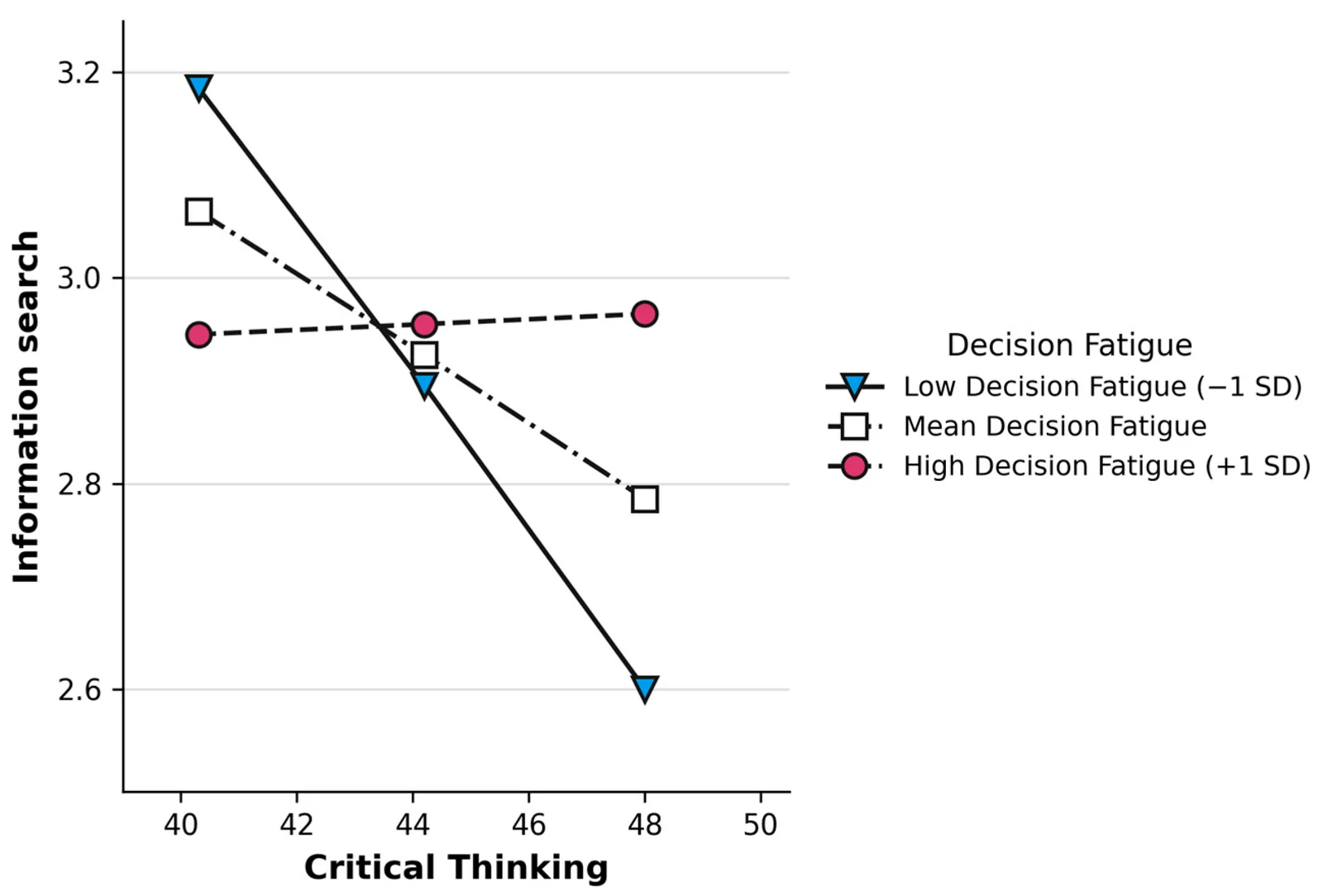
Moderating effect of Decision Fatigue on the relationship between Critical Thinking and passive acceptance of GenAI Output for Information search.

With Information search as the dependent variable, Models 1, 2, and 3 were all statistically significant. In Model 1, age was the only significant predictor. In Model 2, two variables emerged as significant: age and Critical Thinking. Model 3 revealed a significant interaction between Critical Thinking and Decision Fatigue (b = 0.008, SE = 0.003, t = 2.31, *p* = 0.022, ΔR^2^ = 0.017), explaining a total of 6.42% of the variance. This effect remained significant under FDR correction but did not survive Bonferroni or Holm correction (Table 3), and should therefore be regarded as provisional pending replication. The conditional effects analysis (Figure 5) showed that, at below-average levels of Decision Fatigue (b = −0.076, *p* = 0.002) and at average levels of Decision Fatigue (b = −0.037, *p* = 0.041), Critical Thinking was associated with a significant reduction in the passive acceptance of AI-generated output. This effect flattened at above-average levels of Decision Fatigue, such that individuals with both high and low levels of Critical Thinking showed a similar tendency to passively accept AI-generated output.

The model with Text generation as the dependent variable did not identify any statistically significant predictors.

For the model with Idea generation as the dependent variable, age was statistically significant in Models 1, 2, and 3, whereas Critical Thinking emerged as a significant predictor only in Model 3. The final model was statistically significant, explaining 6.04% of the variance (R^2^ = 0.0604, F = 3.09, *p* = 0.006, df = 288). These findings indicate that age is a significant predictor of students’ tendency to passively accept GenAI-generated output, with younger students showing a greater tendency to accept AI-generated output without making personal modifications than older students.

Likelihood-ratio comparisons between the main-effects model (Model 2) and the interaction model (Model 3) reproduced the same task-specific pattern of significant and non-significant interaction effects observed in the hierarchical linear regression models: significant moderation effects for Text revision, Creating quizzes, Receiving feedback, and Information search, and no significant improvement in model fit for Idea generation or Text generation (Appendix A Table A1). These results indicate that the observed pattern of moderation effects does not depend on the choice between linear and ordinal modelling of the outcome variables.

## 4. Discussion

The findings of the present study provide evidence regarding the role of Critical Thinking and Decision Fatigue in the passive acceptance of GenAI-generated output. Before examining how Decision Fatigue moderates the relationship between the variables, it is important to consider the overall role of Critical Thinking in the passive acceptance of GenAI-generated output. Accordingly, the following discussion focuses on the main effects of Critical Thinking, whereas the conditional effects will be addressed subsequently in the context of the moderation analysis (Hypothesis 3). Overall, Critical Thinking showed a consistently negative association across all six academic activities considered (Model 2), reaching statistical significance for Information Search. Taken together, these findings support Hypothesis 1.

In addition to confirming existing evidence in the literature, the findings of the present study suggest that the relationship between critical thinking and AI-mediated learning may not be uniform across different academic activities. Although the association between Critical Thinking and the acceptance of AI-generated output without personal modification showed a consistently negative trend across all six activities considered, statistically significant effects emerged only for Information search. This specific pattern suggests that critical thinking may exert a stronger influence in tasks requiring students to evaluate, select, and integrate information than in tasks primarily involving content production. Consequently, the role of critical thinking in AI-mediated learning may depend not only on individual dispositions but also on the epistemic demands of the task.

These findings are consistent with a growing body of literature identifying critical thinking as a protective resource against unreflective forms of Generative Artificial Intelligence use. Previous studies have reported negative associations between critical thinking and AI dependence (Amaya et al., 2025), showing that students with higher levels of critical thinking are more likely to verify system-generated information, integrate it with their existing knowledge, and maintain control over the final product (Singhachotsukpat et al., 2025; Hou et al., 2025). From this perspective, critical thinking represents a fundamental resource for the informed, reflective, and supervised use of AI in educational contexts (Walter, 2024).

However, the present study extends these findings by showing that the protective effect of critical thinking is not expressed uniformly across different academic activities and appears to depend, at least in part, on the cognitive conditions under which the interaction with AI takes place.

Decision Fatigue did not emerge as a statistically significant direct predictor of the passive acceptance of GenAI-generated output in any of the academic activities considered. Thus, the analyses did not provide evidence of a consistent direct association between Decision Fatigue and passive acceptance across academic tasks, a pattern in line with Hypothesis 2. It is important to note, though, that these nonsignificant findings should not be interpreted as evidence for the absence of an effect. By contrast, the inclusion of the interaction term (Critical Thinking*Decision Fatigue) in the final regression models revealed a different pattern. Specifically, Decision Fatigue moderated the relationship between Critical Thinking and passive acceptance in the activities of Text Revision, Creating Quizzes, Receiving Feedback, and Information Search at the uncorrected significance level. However, correction for multiple comparisons across the six interaction tests (Bonferroni, Holm, and FDR; Table 3) revealed a differentiated pattern of evidence. The moderating effect of Decision Fatigue was robust across all three correction procedures, including the most conservative, for Text revision and Receiving feedback. The interaction effects observed for Information search and Creating quizzes remained significant only under the less conservative FDR procedure; these findings are therefore considered more provisional and warrant replication in independent samples. No moderation effect was observed for Idea generation or Text generation under any correction procedure. These findings indicate that the influence of Decision Fatigue is better understood as a contextual factor that shapes the effectiveness of Critical Thinking—robustly so in activities involving revision and feedback, and more tentatively in activities involving information search and quiz creation—rather than as a uniform, direct predictor of passive GenAI use. In light of these findings, Hypothesis 3 received robust support, consistent across all applied correction procedures, for two of the six academic activities examined, and additional, more provisional support—detected only under the least conservative procedure—for two further activities. The most relevant contribution of the present study concerns the moderating role of Decision Fatigue. Although the existing literature has consistently identified Critical Thinking as a protective factor against unreflective use of AI-generated content, the present findings suggest that this protective function is not expressed uniformly across different cognitive conditions. In other words, possessing a disposition toward critical thinking may represent a necessary, but not sufficient, condition for this resource to be effectively activated and translated into behavior during interactions with Generative Artificial Intelligence systems.

The analyses indicate that, even in the presence of high levels of critical thinking, when individuals experience decision fatigue, the protective function of critical thinking against the passive use of AI is substantially weakened. These findings provide empirical support for the dispositional theories of thinking proposed by Perkins et al. (1993). According to these perspectives, the effective exercise of critical thinking requires more than simply possessing the necessary cognitive abilities; it also requires the willingness and the opportunity to activate those abilities within a specific context. The findings of the present study suggest that Decision Fatigue may represent a condition capable of limiting this activation process. Consequently, students may possess the cognitive resources necessary to critically evaluate AI-generated output, yet lack the mental resources required to mobilize those abilities effectively in a given situation.

Overall, these findings suggest that the relationship between critical thinking disposition and behavior is not stable but may vary according to the cognitive conditions under which individuals interact with AI. From this perspective, the predictive value of critical thinking lies not only in an individual’s dispositional level but also in the actual possibility of activating that disposition when interacting with Generative Artificial Intelligence systems.

A further aspect deserving specific attention concerns the distribution of the moderating effects across the different academic activities examined. It is noteworthy that significant interactions (Hypothesis 3), with varying degrees of robustness to correction for multiple comparisons (see Table 4), emerged primarily in activities involving monitoring, evaluation, verification, and revision processes, such as Information search, Receiving feedback, Text revision, and Creating quizzes.

One possible interpretation is that critical thinking plays a particularly important role when students are required to supervise and evaluate AI-generated output, rather than when AI is used solely as a tool for content production. This interpretation is consistent with recent theoretical perspectives suggesting that Generative Artificial Intelligence shifts cognitive demands away from the direct execution of tasks toward processes of supervision, monitoring, and evaluation (Lee et al., 2025).

A complementary perspective, however, deserves consideration. Rather than reflecting a reduction in critical engagement, the observed pattern may indicate a strategy for allocating cognitive resources. In light of the theory of bounded rationality (Simon, 1955) and resource-rational analysis (Lieder & Griffiths, 2020), students experiencing high levels of Decision Fatigue may deliberately choose to delegate specific cognitive operations to AI systems when the perceived costs of verification outweigh the expected benefits. From this perspective, the acceptance of AI-generated output would not necessarily represent a form of cognitive disengagement but rather a context-dependent decision regarding the management of limited cognitive resources.

Taken together, the findings suggest that the relationship between Critical Thinking and AI-supported learning cannot be fully understood by focusing exclusively on students’ dispositional characteristics. The cognitive conditions under which the interaction takes place appear to play a crucial role in determining whether, and to what extent, critical thinking is effectively translated into behavior. More broadly, the present study contributes to the growing body of literature suggesting that the educational impact of Generative Artificial Intelligence depends not only on the characteristics of the technology itself but also on the cognitive resources that students are both able and willing to mobilize during their interaction with AI-generated content.

Despite its scientific contribution, the present study has several limitations. First, participants were recruited using a non-probability sampling strategy, specifically convenience sampling. A further limitation concerns the use of self-report measures to assess reliance on AI-generated output, personal dispositions (Critical Thinking), and cognitive fatigue (Decision Fatigue). In addition, the internal consistency of the Italian CTDS translation used in the present study (α = ω = 0.69) was slightly below the conventional 0.70 benchmark; although values in the 0.60–0.70 range have been considered acceptable in exploratory research (Hair et al., 2019), this level of reliability may have attenuated the observed regression coefficients, such that the reported associations between Critical Thinking and passive acceptance of AI-generated output should be regarded as conservative estimates of the underlying relationships. Both the CTDS and the DFS Italian versions used in this study are still undergoing formal psychometric validation, and this preliminary status should be taken into account when interpreting the reported associations. Similarly, although the six task-specific items did not undergo formal expert review or pilot testing, their development was informed by previous literature on academic uses of GenAI (Johnston et al., 2024; Stojanov et al., 2024; Wang et al., 2025). Nevertheless, future research should further establish their content validity through systematic expert review and pilot testing. Sample sizes also varied across academic activities (ranging from 205 to 308 participants) due to the task-specific structure of the items, which may have affected the precision of task-specific estimates.

Future research should adopt quasi-experimental designs to directly observe how individuals interact with AI systems. Another limitation lies in the cross-sectional nature of the study, which does not allow the assessment of potential changes over time in users’ interactions with AI. Therefore, future studies should adopt longitudinal designs to examine changes in the ways individuals interact with these systems.

Despite these limitations, the present study offers a novel perspective on the role of personal dispositions in shaping the use of AI-generated output, contributing to a better understanding of this complex interaction and highlighting important educational, instructional, and ethical implications.

## 5. Conclusions

Since 2022, the adoption of artificial intelligence in educational contexts has grown exponentially. The present study examined university students’ reliance on unmodified GenAI-generated output across six academic activities. Six ad hoc, task-specific items were developed to assess students’ reliance on GenAI-generated output across these activities, and their relationship with two dispositional characteristics, Critical Thinking and Decision Fatigue, was examined.

Hierarchical multiple regression was employed to identify the variables that significantly predicted passive acceptance of AI-generated output in each academic activity. Because the six items were designed to capture conceptually and behaviorally distinct academic contexts rather than interchangeable indicators of a single construct, each activity was analyzed as an independent dependent variable, with demographic characteristics (sex, age, and STEM field), Critical Thinking, Decision Fatigue, and the Critical Thinking × Decision Fatigue interaction term entered as predictors.

Critical Thinking was confirmed as a consistently negative predictor of passive acceptance across all six academic activities, reaching statistical significance most clearly for Information search, in support of Hypothesis 1. Decision Fatigue did not emerge as a statistically significant direct predictor in any of the six models, a pattern consistent with Hypothesis 2, although these nonsignificant findings should not be interpreted as evidence for the absence of an effect. The central contribution of the present study concerns the moderating role of Decision Fatigue in the relationship between Critical Thinking and the passive use of AI-generated output (Hypothesis 3). Given that this hypothesis was tested six times, once per academic activity, Bonferroni, Holm, and Benjamini–Hochberg FDR corrections were applied to evaluate the robustness of these interaction effects under procedures of differing stringency. This analysis revealed a differentiated pattern of evidence rather than a uniform one. The moderating effect of Decision Fatigue was found to be robust across all three correction procedures, including the most conservative, for Text revision and Receiving feedback, indicating consistent evidence that the protective role of critical thinking against passive AI reliance is contingent on decision fatigue in these two activities. The interaction effects observed for Information search and Creating quizzes remained significant only under the less conservative FDR procedure and did not survive Bonferroni or Holm correction; these findings are therefore considered more provisional and should be interpreted with caution pending replication in independent samples. No evidence of moderation emerged for Idea generation or Text generation under any correction procedure.

This differentiated pattern is taken to suggest a more specific model: the moderating role of Decision Fatigue appears most robust in academic activities that require sustained monitoring, evaluation, and revision of already-produced content (text revision, feedback reception), and comparatively weaker, though still detectable, in activities involving information retrieval and content verification (information search, quiz creation). This distinction is consistent with theoretical accounts suggesting that generative AI shifts cognitive demands from task execution toward supervisory and evaluative processes, and that these supervisory demands may be particularly vulnerable to depletion under conditions of decision fatigue.

Additional robustness analyses using ordinal logistic regression, considered appropriate given the five-category Likert structure of the outcome variables, reproduced the same task-specific pattern of significant and non-significant interaction effects observed in the hierarchical linear regression models, further supporting the stability of these conclusions across alternative modelling approaches.

Taken together, these findings offer new insight into the boundary conditions under which critical thinking operates as a protective resource against the passive use of generative AI, and underscore that this protective function is not stable but contingent on students’ cognitive state at the moment of interaction. These results support the need to design educational interventions that foster critical thinking dispositions while simultaneously promoting cognitively sustainable learning conditions, particularly in academic activities that demand active supervision and evaluation of AI-generated content. Given the modest proportion of variance explained by the final models (ranging from approximately 6% to 9%) and the cross-sectional, convenience-sample design of the study, these conclusions should be regarded as an initial, theoretically grounded contribution warranting replication with larger and more representative samples, and, where feasible, longitudinal or experimental designs capable of establishing the direction of the observed associations.

## Figures and Tables

**Table 1:** Descriptive statistics for six task-specific items assessing passive acceptance of GenAI output across academic tasks.

Activity	N	Mean	SD	Minimum	Maximum	Skewness	Kurtosis
Idea generation	295	2.57	1.08	1.00	5.00	0.19249	−0.913
Text generation	265	2.06	1.05	1.00	5.00	0.76905	−0.199
Text revision	258	2.75	1.20	1.00	5.00	0.01546	−1.066
Creating quizzes	212	3.15	1.30	1.00	5.00	−0.19253	−1.110
Receiving feedback	229	2.97	1.20	1.00	5.00	−0.05612	−1.021
Information search	308	2.89	1.20	1.00	5.00	−0.07919	−0.969

**Table 2:** Hierarchical regressions.

Models	Text Revision	Creating Quizzes	Receiving Feedback	Information Search	Idea Generation
**Model 1**					
Gender	0.003	0.409	−0.045	−0.037	0.091
Age	−0.005	−0.113 ***	−0.057	−0.083 **	−0.068 **
STEM	0.112	−0.156	0.064	0.202	−0.236
R^2^	0.002	0.064 **	0.014	0.033 *	0.029 *
**Model 2**					
Gender	0.033	0.398	−0.046	−0.027	0.133
Age	0.003	−0.109 **	−0.048	−0.072 *	−0.055 *
STEM	0.090	−0.181	0.014	0.150	−0.297
Critical Thinking	−0.007	−0.023	−0.030	−0.038 *	−0.032
Decision Fatigue	0.023	−0.002	−1.72 × 10^−4^	0.003	0.019
R^2^	0.13	0.068 *	0.023	0.048 *	0.052 **
**Model 3**					
Gender	0.057	0.402	−0.051	−0.018	0.148
Age	−0.004	−0.113 **	−0.057	−0.074 **	−0.055 *
STEM	0.027	−0.243	−0.006	0.126	−0.318
Critical Thinking	−0.153 ***	−0.131 *	−0.155 ***	−0.121 **	−0.086 *
Decision Fatigue	−0.567 ***	−0.426 *	−0.495 **	−0.335 *	−0.195
Critical Thinking × Decision Fatigue	0.013 ***	0.010 *	0.011 **	0.008 *	0.005
R^2^	0.067 **	0.090 **	0.063 *	0.064 **	0.0604 **

Note. * *p* < 0.05, ** *p* < 0.01, *** *p* < 0.001. Text generation is not included in this table because no predictor reached statistical significance in Models 1–3 for this outcome; full results for Text generation are reported in the text (Section 3).

**Table 3:** Interaction term (Critical Thinking × Decision Fatigue) *p*-values under three multiple comparison correction procedures.

Academic Activity	*p* (Uncorrected)	*p* (Bonferroni)	*p* (Holm)	*p* (FDR-BH)
**Text revision**	<0.001	0.006	0.006	0.006
**Receiving feedback**	0.002	0.012	0.010	0.006
**Information search**	0.022	0.132	0.088	0.042
**Creating quizzes**	0.028	0.168	0.088	0.042
**Idea generation**	0.113	0.678	0.226	0.136
**Text generation**	0.367	1.000	0.367	0.367

**Table 4:** Conditional effects of Critical Thinking on passive acceptance of GenAI output across academic tasks at different levels of Decision Fatigue.

Dependent Variable	Low Decision Fatigue	Mean Decision Fatigue	High Decision Fatigue
**Text revision**			
Effect	−0.072 **	−0.003	0.067 *
t	−2.805	−0.131	2.437
95% CI	[−0.122, −0.021]	[−0.040, 0.035]	[0.013, 0.120]
**Creating quizzes**			
Effect	−0.073 *	−0.025	0.024
t	−2.235	1.047	0.759
95% CI	[−0.138, −0.009]	[−0.071, 0.022]	[−0.038, 0.086]
**Receiving feedback**			
Effect	−0.086 **	−0.027	0.032
t	−3.117	−1.302	1.117
95% CI	[−0.139, −0.031]	[−0.067, 0.014]	[−0.025, −0.088]
**Information search**			
Effect	−0.076 **	−0.037 *	0.002
t	−3.106	−2.050	0.088
95% CI	[−0.124, −0.023]	[−0.072, −0.002]	[−0.047, 0.051]

Note. * *p* < 0.05, ** *p* < 0.01.

## Data Availability

The raw data supporting the conclusions of this article will be made available by the authors on request.

## Abbreviations

The following abbreviations are used in this manuscript:

AI	Artificial Intelligence
CT	Critical Thinking
DF	Decision Fatigue
GenAI	Generative Artificial Intelligence

## Appendix A

**Table A1:** Hierarchical linear regression (HLR) and ordinal logistic regression (OLR) results for the Critical Thinking × Decision Fatigue interaction term.

Academic Activity	HLR (Interaction *p*)	OLR (LR Test M2 vs. M3)	OLR (Wald Test)	Interaction Retained?
Idea generation	0.113	0.120	0.003	No
Text generation	0.367	0.381	0.116	No
Text revision	<0.001	<0.001	<0.001	Yes
Creating quizzes	0.028	0.034	<0.001	Yes
Receiving feedback	0.002	0.002	<0.001	Yes
Information search	0.022	0.017	<0.001	Yes

Note. HLR *p*-values correspond to the interaction term in Model 3 of the hierarchical linear regression. OLR results are based on likelihood-ratio comparisons between Model 2 (main effects only) and Model 3 (main effects + interaction); Wald test *p*-values for the interaction coefficient are reported for completeness. A discrepancy emerged only for Idea generation, where the interaction coefficient was significant according to the Wald test (*p* = 0.003) but the likelihood-ratio test for overall model improvement was not (*p* = 0.120); the likelihood-ratio test was considered the principal criterion for interpretation, consistent with the model-comparison logic adopted in the linear regression analyses.

## Appendix B

**Table A2:** Distribution of participants by university of affiliation.

University	*n*	%	University	*n*	%
IUSVE	162	42.3	Ca’ Foscari University of Venice	4	1.0
University of Padua	37	9.7	Marche Polytechnic University	4	1.0
University of Milano-Bicocca	23	6.0	IUAV University of Venice	3	0.8
University of Udine	17	4.4	University of Bergamo	3	0.8
University of Bologna	12	3.1	University of Siena	3	0.8
University of Trento	12	3.1	Sapienza University of Rome	2	0.5
University of Perugia	11	2.9	University of Messina	2	0.5
University of Camerino	11	2.9	eCampus University	2	0.5
University of Milan	9	2.3	Università Cattolica del Sacro Cuore	1	0.3
University of Bari Aldo Moro	8	2.1	University of Verona	1	0.3
Roma Tre University	8	2.1	University of Pavia	1	0.3
University of Turin	6	1.6	University of Pisa	1	0.3
University of Parma	6	1.6	University of Tuscia	1	0.3
University of Florence	6	1.6	University of Trieste	1	0.3
University of Ferrara	6	1.6	IUL–Italian University Line	1	0.3
University of L’Aquila	6	1.6	Universitas Mercatorum	1	0.3
University of Modena and Reggio Emilia	6	1.6	University of Catania	1	0.3
University of Cagliari	5	1.3			
**Total**				**383**	**100.0**
